# Size‐dependent mating pattern in a nuptial gift‐giving insect

**DOI:** 10.1002/ece3.4763

**Published:** 2018-12-06

**Authors:** Martina Dorková, Ladislav Naďo, Benjamín Jarčuška, Peter Kaňuch

**Affiliations:** ^1^ Institute of Forest Ecology Slovak Academy of Sciences Zvolen Slovakia; ^2^ Faculty of Ecology and Environmental Sciences Technical University in Zvolen Zvolen Slovakia

**Keywords:** katydid, phenotype, polyandry, sexual preferences

## Abstract

The reproductive interests of females and males often diverge in terms of the number of mating partners, an individual’s phenotype, origin, genes, and parental investment. This conflict may lead to a variety of sex‐specific adaptations and also affect mate choice in both sexes. We conducted an experiment with the bush‐cricket *Pholidoptera griseoaptera* (Orthoptera, Tettigoniidae), a species in which females receive direct nutritional benefits during mating. Mated individuals could be assigned due to the genotype of male spermatodoses, which are stored in the female’s spermatheca. After 3 weeks of possible copulations in established mating groups which were random replications with four females and males we did not find consistent assortative mating preference regarding to body size of mates. However, our results showed that the frequency of within‐pair copulations (192 analyzed mating events in 128 possible pairwise combinations) was positively associated with the body size of both mated individuals with significant interaction between sexes (having one mate very large, association between body size and the number of copulations has weaken). Larger individuals also showed a higher degree of polygamy. This suggests that body size of this nuptial gift‐giving insect species is an important sexual trait according to which both sexes choose their optimal mating partner.

## 1 INTRODUCTION

Choosing an optimal partner for mating is a problem of an extraordinary complexity. No wonder that sexual selection has been the focus of attention of evolutionary biologists for more than a century (Andersson, 1994; Arnqvist & Rowe, 2005; Fisher, 1915; Taylor, Price, & Wedell, 2014; Trivers, 1972). In light of recent research it must be acknowledged that both females and males try to choose the best individual(s) out of a number of competing mates (review by Edward & Chapman, 2011). Moreover, in many cases it is not enough to convince or force a mate into copulation but also to invest its time and energy in offspring (Gwynne, 1984a, 1986, 1990; Simmons & Gwynne, 1993; Trivers, 1972; Vahed, 2007b). For both sexes which should maximize their reproductive potential the courtship time is relatively short and comprehensive knowledge about the potential mate is impossible. One of the well‐known characteristics of ectotherms considered to be relevant in the choice of a sexual partner is the body size (Engqvist & Sauer, 2001; Gilburn, Foster, & Day, 1992; Partridge, Hoffmann, & Jones, 1987). This attribute of selection plays an important role in many species, since the reproductive success of most invertebrates correlates positively with the size of the female’s body (Honěk, 1993). In male insects with a promiscuous mating system, size‐assortative mating is characteristic because phenotypic fertility indicators are crucial in male choice as larger or heavier females may contain more mature eggs (Bonduriansky, 2001; Honěk, 1993; Katvala & Kaitala, 2001), while larger males are also more successful in courting or defending larger females (Clutton‐Brock, 2007; Jiang, Bolnick, & Kirkpatrick, 2013).

Mating with more mates is a phenomenon in a wide range of species (Arnqvist & Nilsson, 2000; Jennions & Petrie, 2000; Taylor et al., 2014; Zeh & Zeh, 2001). Females have been shown to gain fitness benefits by preferring superior males that indirectly indicate their genetic quality (Andersson, 1994; Jennions & Petrie, 2000; Roberts, Hale, & Petrie, 2006; Tregenza & Wedell, 2000). Females may increase their own reproductive success using phenotypic traits to choose mates with “good genes” (Mays & Hill, 2004; Trivers, 1972). The degree of genetic dissimilarity between potential mates as another category of genetic influence is thought to be central to the evolution of polygamy (Taylor et al., 2014; Tregenza & Wedell, 2000; Zeh & Zeh, 2003). Multiple mating can serve to prevent inbreeding (Penn & Potts, 1999) and increase heterozygosity in offspring, with consequent indirect fitness benefits to parents (Yasui, 1998). Therefore, preferences toward heterozygosity may thus be another adaptation that favors the production of diverse and superior competitors (Brown, 1997; Roberts et al., 2006).

Possible assortative mating of individuals with similar body size can be even less important if the reason for multiple mating lies in the direct nutritional benefits that a female gains from multiple copulations in species that have nuptial feeding (Arnqvist & Nilsson, 2000; Simmons, 1990; Vahed, 1998; Wagner, 2011). Bush‐crickets (Orthoptera: Tettigoniidae) provide unique opportunities to test various hypotheses on mechanisms behind entangled mating patterns. Their nuptial gifts are produced and transferred by males during copulation in the form of a nutritious spermatophylax attached to the sperm‐containing ampulla, both comprising a spermatophore (Gwynne, 1995, 1997; Vahed, 2007a, 2007b; Wedell & Ritchie, 2004). These gifts confer considerable benefits to females, and heavier males that provide larger spermatophylax meals are generally more preferred by females (Gwynne, 1982; Gwynne, Brown, & Codd, 1984; Lehmann & Lehmann, 2008; Wedell & Sandberg, 1995). The bush‐cricket mating system cannot be considered as a simple collaboration between the sexes, with females receiving a nutritional substance and males protecting their ejaculates (Gwynne, 2001). However, it is a permanent sexual conflict in which the sexes have different demands for spermatophore quality and size (Arnqvist & Rowe, 2005; Gwynne, 2008; Parker, 2006; Vahed & Gilbert, 1996; Vahed, 2007a). The relative amount of sperm received from the male determines the proportion of eggs that are fertilized (Parker, Simmons, & Kirk, 1990), while this antagonistic coevolution is even more entangled, because males also transfer in spermatophylax substances that may manipulate female behavior via inhibition of further copulation or successful sperm transfer (Gwynne, 1986; Lehmann et al., 2018; Vahed, 2006). The mating potential of males thus plays an important role, because if a male has fertilized more females, its reproductive success would be several times higher than the success of each fertilized female (Wedell, Gage, & Parker, 2002). Since other males have the same reproductive interests, they also attempt to fertilize as many females as possible, and there is an energy‐demanding competition between them (Simmons, 2005). However, because a female acquires sperm from several males and collects them inside the reproductive apparatus (spermatheca) in the form of spermatodoses for the rest of its life, sperm competition for egg fertilization keeps on going (Hockham, Graves, & Ritchie, 2004; Parker, Zaborowska, Ritchie, & Vahed, 2017; Simmons, 2005; Wedell et al., 2002).

The size of the spermatodoses could be accepted as a proxy for ejaculate volume and likely also as the overall spermatophore size (Parker et al., 2017; Vahed, 2003; Vahed & Gilbert, 1996). Although females should prefer larger males, spermatophore production is energy‐consuming (Gwynne, 1984a; Simmons, 1990, 1993) and a negative association has been found among‐ and within‐species between spermatodose size and mating rate in bush‐crickets (Jarčuška & Kaňuch, 2014; Vahed, 2006). We therefore assumed a conflict concerning optimal body size if multiple copulations with nuptial gifts occur. However, knowledge about within‐pair mating frequency (i.e., the number of copulations that are associated with a particular male and female) with respect to individuals’ phenotypes in some highly polygamous system is missing. Using an experimental laboratory setup that allowed pair assignment of each copulation and to estimate female’s benefits from and males’ investments into copulations, we aimed in our study (a) to analyze the frequency of individual copulations and the degree of polygamy of each sex, (b) to test size‐assortative mating, and (c) to model the number of within‐pair copulations in relation to the phenotype of mating partners in the dark bush‐cricket *Pholidoptera griseoaptera*.

## 2 MATERIALS AND METHODS

### 2.1 Study species and mating system

**Figure 1:**
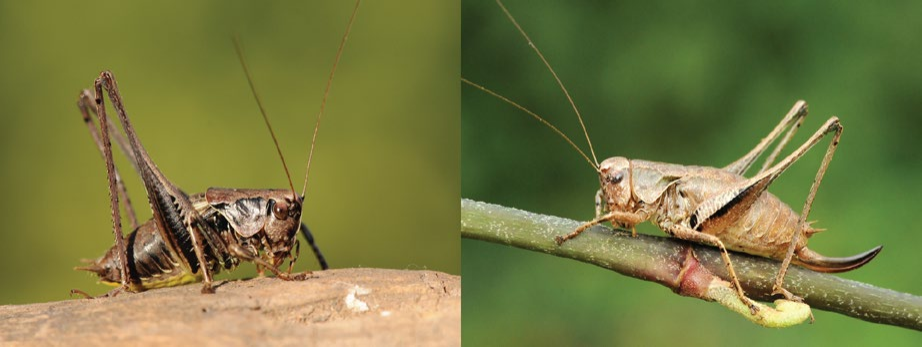
Adult male (left) and female (right) of the dark bush‐cricket, *Pholidoptera griseoaptera* in Slovakia. Photographs by J. Svetlík

The dark bush‐cricket, *P*.* griseoaptera*, is flightless ground‐dwelling species (Figure 1) occurring throughout Europe, except for the most northern, western, and southern regions (Hochkirch et al., 2016). The preferred habitats of this widespread and abundant bush‐cricket are forest edges, clearings, open woodland and hedgerows, gardens, mainly at altitudes from 100 to 1,400 m a.s.l., rarely up to 2,600 m a.s.l. in the Alps (Zuna‐Kratky et al., 2017). Females lay eggs in bark or rotten wood, and nymphs are hatched often after two winter diapauses. Usually in July, after the last molting, nymphs become adults capable of reproduction. The male’s stridulation plays a crucial role in the search for a potential partner. The audibility can reach about 10 m with a frequency range of 10–40 kHz, while males stridulate even in rainy weather at low temperatures until the first signs of frost in late autumn (Zuna‐Kratky et al., 2017). Although the female is attracted to the intense sound of the male, the final decision on the course of mating is in the female’s direction (Gwynne, 2001). A male may be accepted or refused depending on the female’s preferences, which have not yet been sufficiently elucidated. If a male is refused, it tries to attract another female (Parker & Simmons, 1989). Prior to copulation, either a male approaches under the female’s abdomen or the female climbs on the male’s back, while during the copulation the male grasps the female’s ovipositor with its cerci or legs (Lehmann & Lehmann, 2008; Vahed, 2007a, 2007b). Copulation may take as long as 40 min by observation and afterward, the male attaches to the female’s genital opening a spermatophylax (gelatinous mass), which is a product of the male’s accessory glands (Vahed, 1998).

### 2.2 Lab rearing of insects

Bush‐crickets nymphs of first or second instars were caught by entomology sweep nets at two sites in central Slovakia during late May and early June 2013. The sites differed according to altitude (170 and 1,180 m a.s.l.) to control for the environmentally driven variability in mating frequency that was found in this species (Kaňuch, Jarčuška, Kovács, & Krištín, 2015). Juvenile individuals were transferred into the lab and reared in glass containers (40 × 20 × 20 cm) with wire netting on top. Bush‐crickets were kept in containers with up to 20 nymphs and fed ad libitum with fresh leaves of the European blackberry (*Rubus fruticosus*), ground dry cat food, oat flakes and special food for crickets which also supplied vitamins (JBL TerraCrick). The containers’ interior was sprayed with water every second day (usually in morning) to provide moisture for safe molting. Light and heat were provided by 25 W daylight lamps with a neodymium sleeve (Exo‐Terra Day Glo) placed over the containers with an L/D cycle of 12/12 hr. The light spectrum of the lamp concentrated at a wavelength of 700–900 nm (UV‐A) contributed to the insects’ physiological well‐being in the rearing facility, which was placed in the basement with some dim natural light penetrating because of the small north‐facing windows. To ensure that virginal adults were being raised, the nymphs were separated by sex in their third or fourth instar. Immediately after full insect development, we established four mating groups for each population, which consisted of randomly selected adults of four females and four males. Altogether 64 individuals in eight groups (128 possible pairwise combinations) were allowed to copulate for 21 consecutive days. It was found that the median mating frequency observed during this period in such lab setting is similar to the field conditions during most of the species lifetime, and thus one can expect that the number of potential mates is also similar to the number that is likely to be encountered under natural conditions (cf. Jarčuška & Kaňuch, 2014, Kaňuch et al., 2015; Vahed, 2006). At the end of experiment, individuals were stored in 98% ethanol for further morphological measurements and genotyping.

### 2.3 Body size and spermatodoses

Using a magnifying glass and a digital calliper (accuracy ± 0.03 mm) we measured the net body length (i.e., without the ovipositor in females and without the cerci in males) in all the adult individuals. We employed this as a commonly used measure of body size in Orthoptera, and as expected, a sexual trait that can be important for mate choice (Andersson, 1994; Brown, 2008; Whitman, 2008). According to the previous knowledge about copulatory system of *P. griseoaptera* and related bush‐crickets from the sub‐family Tettigoniinae, we assumed that each spermatodose represents one copulation and vice versa (Gwynne, 2001; Vahed, 2006). In order to estimate the female’s benefits from and the males’ investments into copulations we measured the diameter of spermatodoses found in females’ spermatheca. The spermatheca was dissected in Ringer’s solution under a 16× binocular enhancer in a Petri dish. The diameter of removed spermatodoses was measured on digital images taken through a binocular enhancer with an image resolution of 100 pixels per mm using GIMP 2.8.10 (GNU Image Manipulation Program; http://www.gimp.org/) and subsequently used to calculate the approximate volume of a sphere in mm^3^.

### 2.4 Genotyping and relatedness

The frequency of within‐pair copulations of each possible pairwise combination in the random mating group was estimated by the number of spermatodoses assigned to respective male that were found in a female spermatheca. This assignment was based on agreement between genotypes of males and spermatodoses. Each female was genotyped as well to control for the possible transfer of the female’s cells from the spermatheca, which may contaminate the sample. To obtain a genetic profile of the adult individuals, we took muscle tissue from the hind femur, while the entire spermatodoses mass was used for that purpose. DNA extraction was conducted according to the so‐called salting‐out protocol (Aljanabi & Martinez, 1997) modified by added RNaseA (Hornett & Wheat, 2012). Six polymorphic microsatellite loci were used for genotyping the material (WPG2‐16, WPG9‐1, WPG1‐28, WPG2‐15, WPG2‐39, WPG7‐11; Arens et al., 2005) according to multiplex PCR protocol (Kaňuch et al., 2012). For DNA template amplification, we added either 1 μl of DNA (20–40 ng/μl) from muscle tissue or 3 μl of DNA (5–10 ng/μl) from spermatodoses into the PCR mastermix. Microsatellite loci were amplified in touchdown PCR (thermocycler Biometra TAdvanced) at the following steps: initial denaturation at 95°C for 5 min, 10 cycles of denaturation at 95°C for 30 s, annealing with gradually decreased temperature at 70–61°C for 30 s and extension at 72°C for 90 s, 25 cycles of the same steps but constant annealing at 60°C and a final elongation step at 72°C for 5 min. The fluorescent labeled PCR products were separated by capillary electrophoresis in an ABI 3730XL genetic analyzer and fragment lengths, estimated relative to the size standard LIZ600, were determined using Geneious 7.0.5 software (Biomatters).

Since there is a known potential for tradeoffs between relatedness and heterozygosity as a trait indicating “good‐genes” in mate‐choice decision, we controlled for this effect by pairwise calculation of relatedness. The Milligan’s likelihood estimator *r*_M_ (Milligan, 2003) was used to determine relatedness between possible pairs in ML‐Relate software, which also accounted for null alleles (Kalinowski, Wagner, & Taper, 2006).

### 2.5 Data analysis

To test assortative mating of individuals, we correlated body size of mates using Pearson’s correlation coefficient. This correlation was performed also for each of two populations separately. For modeling the frequency of within‐pair copulations (response variable) we employed from the family of regression models two random intercept, mixed‐effects models with a Poisson error distribution, log‐link function, and type II SS. The population origin, relatedness and female and male body sizes were used as fixed factors in construction of a model with main effects only and also in an optimal (i.e., minimal adequate) model that used a so‐called top‐down strategy when removing non‐significant interactions from the full model (Zuur, Ieno, Walker, Saveliev, & Smith, 2009). These models were compared and ranked using the Akaike information criterion (AIC). Because the mating of individuals was restricted to assigned mating groups, the mating group was used as a random factor to account for possible auto‐correlation between the data from the same group in both models. Model parameters were estimated using the function lmer with Laplace approximation in the package “lme4” 1.1‐15 (Bates, Maechler, Bolker, & Walker, 2015) of the R 3.4.4 software (R Core Team, 2018). The variance explained by all variables in the model (conditional *R* ^2^) and by its fixed factors only (marginal *R* ^2^) was computed using the R‐package “MuMIn” 1.40.4 (Bartoń, 2018). The relationship between the total volume of spermatodoses of a respective male in a female spermatheca (response variable) and the number of within‐pair copulations was explored using simple linear regression.

## 3 RESULTS

**Figure 2:**
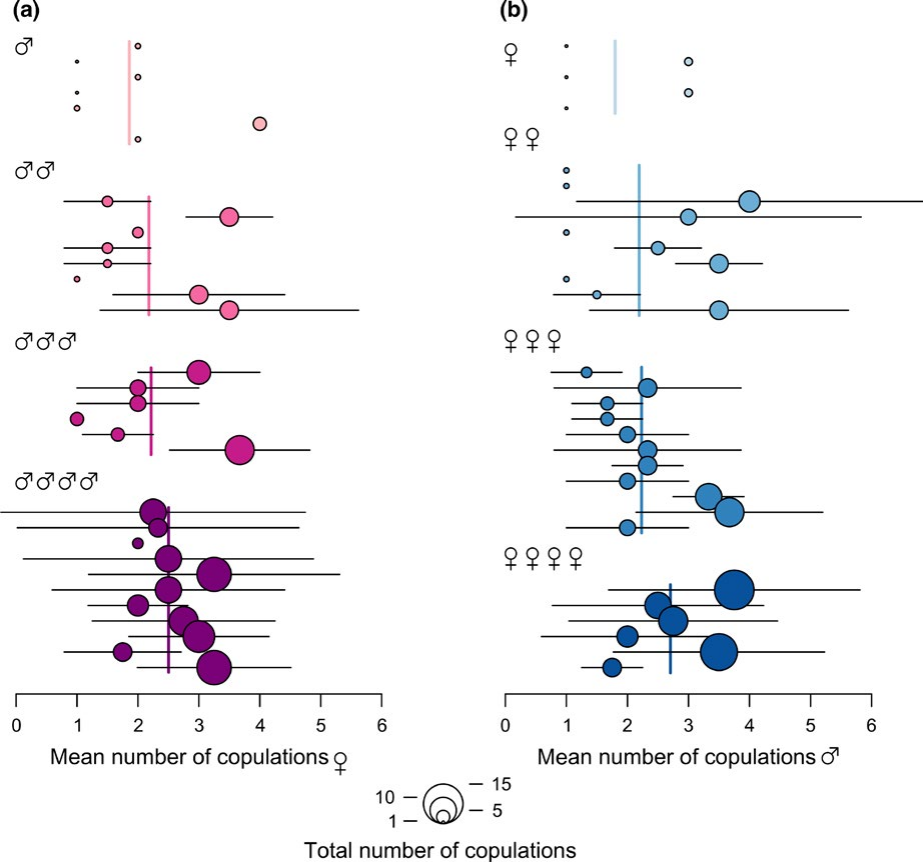
Forest plot of the mean number of copulations of (a) females and (b) males of *Pholidoptera griseoaptera* in established random mating groups (four females and four males in each). Circles represent the mean number of copulations (±*SD* as horizontal segments) of each individual per number of its mates and have areas proportional to the total number of copulations executed by this individual. Individuals are ordered according to the number of their mates (ascending from single mate to four mates)

Altogether we recorded 199 mating events between 32 males and 32 females of *P. griseoaptera* that were allowed to assemble 128 possible combinations in randomly established mating groups. Genotyping of spermatodoses failed in seven cases, thus 192 mating events were analyzed. The median number of individual copulations in both sexes was six (range females, 1–13; males 1–15). Variability of the promiscuity level was relatively large; seven females and five males copulated with one mate only, and in contrast, 11 females (34%) and six males (19%) performed copulations with all four available mates in the mating group (Figure 2).

**Figure 3:**
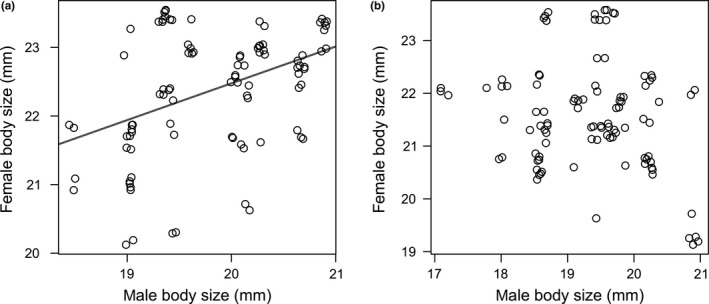
Correlations of sizes of male and female mates in two different populations: (a) low altitude population, (b) high altitude population. Solid line shows significant size‐assortative mating pattern. Overlapping data points are plotted with jitter to enable better visualization

**Figure 4:**
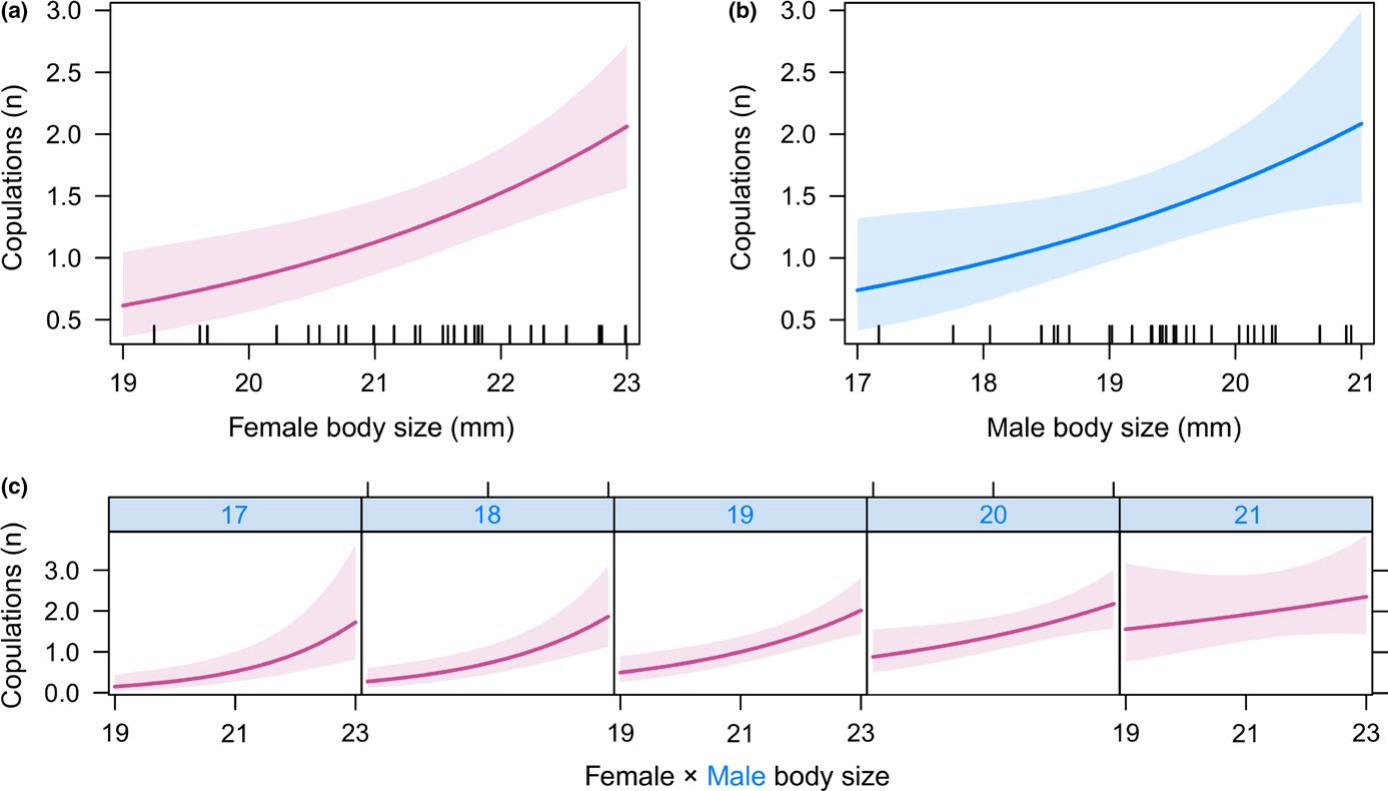
The effects of (a) female and (b) male body size on the number of within‐pair copulations and (c) the interaction effect of female size (values in *x*‐axis) with regard to specific male size (values in headers). Prediction lines (with 95% confidence intervals) are derived from the random intercept mixed‐effects model (Table 1)

If we did not consider frequency of within‐pair copulations, we did not observe consistent size‐assortative mating across different populations (Pearson’s *r* = 0.09; *t* = 0.82, *df* = 80, *p* = 0.41). There was a significant positive correlation between female’s and male’s body size in pairs from the lower altitude population (*r* = 0.33, *t* = 2.12, *df* = 38, *p* = 0.040), while nonsignificant pattern was found in the higher altitude population (*r* = −0.22; *t* = −1.47, *df* = 40, *p* = 0.14; Figure 3). However, the number of within‐pair copulations ranged considerably from one to six, while 46 possible pairwise combinations of males and females (36%) did not copulate during the 3 weeks of the experiment. Both male and female body size had statistically significant effects on the mean number of within‐pair copulations, whereas interaction of these variables also had a significant effect in the minimal adequate model (Figure 4, Table 1). Neither the tested population origin nor relatedness had an effect on the mean number of within‐pair copulations. Overall, the mean pairwise relatedness was very low, *r*_M_ = 0.035 (range 0–0.540). Fixed factors explained 15% (*R* ^2^_m_) of the data variability in the model with the main effects only and 20% in the minimal adequate model; however, another 1% and 3% (*R* ^2^_c_ – *R* ^2^_m_) could be explained by the mating group as a random factor in these models, respectively. Comparing *χ* ^2^ values of terms in these mixed‐effects models, the effect of female size appears stronger than of male (Table 1). Both models seem to be valid because the second‐order ranked model, here the model with additive effects of fixed factors without interactions, was also well supported (ΔAIC = 1.5). This shows that frequent within‐pair copulations were likely to occur in pairs that comprised the largest available individuals in the mating groups; however, having one mate very large, association between body size and the number of copulations has weaken (Figure 4c).

**Table 1:** Results of random intercept mixed‐effects models constructed either with the main effects only or as the minimal adequate one. The frequency of within‐pair copulations (the number of spermatodoses assigned to respective male that were found in a female spermatheca as a response variable) in pairs of *Pholidoptera griseoaptera* is explained by three fixed factors and their significant second‐order interaction, respectively. Mating group was used as the random factor

Effect	*χ* ^2^	*df*	*p*
Main effects only			
Population	3.21	1	0.073
Relatedness	0.01	1	0.92
Female body size	12.72	1	<0.001
Male body size	5.97	1	0.015
Minimal adequate			
Female body size	10.99	1	<0.001
Male body size	5.99	1	0.014
Female × Male body size	5.40	1	0.020

On the other hand, the total volume of spermatodoses increased significantly with the number of copulations executed by a pair (*R* ^2^_adj._ = 0.87, *p* < 0.001) what suggested positive relationship between multiple mating and possible benefits from or investments into copulations, respectively. Thus, the mean increase in spermatodoses (= ejaculate) volume was 0.35 (*SE* ±0.07) mm^3^ for each within‐pair copulation.

## 4 DISCUSSION

Our experiment shows that mating pattern in *P. griseoaptera* was associated with the phenotype of both females and males, though we did not find consistent assortative mating preference of this bush‐cricket species because body size of mates from the higher altitude population did not show statistically significant correlation. Nevertheless, the mean correlation coefficient of positive size‐assortative mating that was found across wide range of different animal taxa (*r* = 0.28; Jiang et al., 2013) is similar to our results from the lower altitude population. Occurrence of such assortative mating is often explained by adaptive evolution through direct or indirect selection on mate choice (Jiang et al., 2013). However, varying intensity of sexual competition among populations (cf. Harari, Handler, & Landolt, 1999; Jiang et al., 2013) suggests an explanation of variation in size‐related assortment in our study. Assortative mating is stronger under high population densities because competition among males for large females is stronger (Jiang et al., 2013; McLain & Boromisa, 1987), while association may be reduced when the costs of choice are high, e.g., due to presence of predators or other environmental constraints (Jennions & Petrie, 1997; Taborsky, Guyer, & Taborsky, 2009). Thus, nonsignificant association between body sizes of mates observed in the higher altitude population might be explained by lower population densities and/or harsher mountainous environment associated with shorter vegetation period.

On the other hand, positive relationship between the body size and the number of within‐pair copulations has shown size‐dependent sexual competition in both tested populations. This suggests competitive potential of sexes as larger females are more fecund and larger males produce more sperms or larger nuptial gifts (cf. Katvala & Kaitala, 2001; Vahed & Gilbert, 1996; Wedell & Sandberg, 1995). Because the largest males and females of *P. griseoaptera* copulated with the same mate several times regardless of its size, the pattern might be also explained through their highest capacity to produce and storage sperms and/or to outcompete smaller individuals of the same sex. As the sex ratio in lab containers has been equal we are not able to distinguish whether individuals coupled because of mate choice or just because of instant availability of a mate. Thus other selection mechanisms behind mating preferences could also affect the observed pattern, but new experimental settings should be established to exclude confounding factors.

Parental investment in nuptial gift‐giving insects is generally larger in females, which must allocate substantial resources related with egg development and oviposition (Gwynne, 1984a, 1984b, 1990; Simmons & Gwynne, 1993). Thus, females should make a principal decision and choose a mate which is able to provide some genetic and nutritional advantages (Gwynne et al., 1984; Jennions & Petrie, 2000; Lehmann, 2012; Wagner, 2011), while females’ preferences for certain male traits may be inherited (Prokop, Michalczyk, Drobniak, Herdegen, & Radwan, 2012). In our case larger body size can reflect an ability of a bush‐cricket male to obtain resources necessary for body development and also an ability to successfully escape from predators because larger size makes it more visible and thus more vulnerable. Alternatively, larger body size may indicate a higher resistance against pathogens (Simmons, 1993). In particular, larger body size of males positively correlates with amount of ejaculate and nuptial gift which is a valuable resource of proteins that replaces the energetic loss related with copulation of a female (Gwynne, 1982; Gwynne et al., 1984; Vahed, 1998) but the total volume of ejaculate and likely also the mean mass of nuptial gifts (see Vahed & Gilbert, 1996) depended on the frequency a within‐pair copulation in our experiment.

On the other hand, females should somehow resist large spermatophores, because they have fitness costs via dose‐dependent inhibition of their receptivity to re‐mate due to highly specialized chemical substances located in the spermatophylax (Gwynne, 1986; Lehmann & Lehmann, 2000; Wedell, 1993). Moreover, after mating the female enters into a refractory period, during which it is not available to another copulation attempt (Lehmann & Lehmann, 2007). Therefore, the loss of proteins and other substances used to produce the spermatophore reduces the male’s fitness, and thus each copulation will limit the potential for further sexual activity of a male (Wedell, 1991). Then copulation with a larger female also bears a risk that the female will be inseminated with yet other male(s) that in turn will reduce the potential for higher paternity of a male. When males of *P. griseoaptera* were experimentally fed by protein less enriched food, they starved and died untimely during the mating period (own unpublished data). Thus the sperm mass transferred during multiple copulations is not alone sufficient, and in such a case sperm competition should likely play a further role in ensuring paternity (cf. Hockham et al., 2004; Simmons, 2005; Vahed & Parker, 2012; Wedell, 1991).

Because individuals of *P. griseoaptera* preferred a similar phenotype for copulation, one could suppose that the presented pattern is a form of assortative rather than random mating. This suggests that the body size of this nuptial gift‐giving species is an important sexual trait in such an antagonistic and highly polygamous system of reproduction, according to which both sexes choose their optimal mating partner. In conclusion, we are still far from disentangling and understanding mating patterns of nuptial gift‐giving bush‐crickets, in particular when it comes to other drivers, such as environmental change, disruption of gene flow, immigration or other demographic events (Kaňuch et al., 2015, 2012) and another adaptive or plastic responses in behavior are likely.

## CONFLICT OF INTEREST

None declared.

## AUTHOR CONTRIBUTIONS

P.K. and B.J. conceived the idea and designed the study; M.D. conducted molecular work and B.J. measured spermatodoses; L.N. and P.K. performed the statistical analyses; and M.D., B.J. and P.K. wrote the manuscript.

## DATA ACCESSIBILITY

Morphological data on body size and spermatodoses and microsatellite genotypes are available from the Dryad Digital Repository: https://doi.org/10.5061/dryad.k4r4927.
